# Help-seeking trajectory in patients with rheumatoid arthritis

**DOI:** 10.1007/s10067-015-3013-z

**Published:** 2015-07-23

**Authors:** Ingris Pelaez, Claudia Infante, Rosana Quintana

**Affiliations:** Rheumatology Service, General Hospital of Mexico, Mexico City, Mexico; Institute for Social Research, UNAM, National Autonomous University of Mexico, Circuito Mario de la Cueva S/N, Ciudad Universitaria, C.P. 04510 Mexico City, Mexico; Provincial Hospital of Rosario, Rosario, Santa Fe Argentina

**Keywords:** Accessibility, Delay, Health care system, Help-seeking, Illness trajectory, Latin American, Rheumatoid arthritis

## Abstract

Early diagnosis and treatment of rheumatoid arthritis (RA) depends on the degree of fit between the characteristics of the patients and those of the health services. Ensuring timely assessment and treatment is the ideal medical care of RA. The reasons that underlay delays and the help-seeking trajectories are contextually determined. This study aims to identify the empirical evidence related to the help-seeking process and delay in RA in Latin America and to create a comprehensive model integrating the RA medical care processes of help-seeking and delay in a mixed health care system with variable accessibility. Non-systematic literature review of studies with both quantitative and qualitative methodology was conducted. Most of the research about delay and its associated variables have been undertaken in European countries and with White population and cannot be translated to the Latin America context where this research is almost inexistent. These countries have a completely different social context, and for most of the population, the health services are insufficient, inaccessible, fragmented, limited, and inequitable. Our results also show that in RA medical care utilization research, the theories and measurements of the constructs of illness trajectories, help-seeking, and accessibility are not integrated. We offer a conceptual framework that integrates help-seeking trajectories, delay, and accessibility of RA medical health services. If research on RA service utilization is to be undertaken in these countries, there is a need for a comprehensive framework than can enable researchers to integrate and contextualize the study of the problems within broad theoretical and methodological perspectives.

## Introduction

Early diagnosis and treatment of rheumatoid arthritis (RA) depends on the degree of fit between the characteristics of the patients and those of the health services. It has been broadly demonstrated that a treatment delay of more than 3 months increases the risk of adverse outcomes [1–7]. In turn, these outcomes are related to devastating impacts on the individual and at different social levels. Research on the factors involved in the delayed seeking of medical attention is highly warranted. Most of the research about delay and its associated variables has been undertaken in European countries and Canada and with White population [8–15].

Research on delay and medical care utilization of RA is almost inexistent in Latin America. Most of the existing research results documenting the problems of delay, help-seeking process, and medical care cannot be translated to developing countries because they describe problems grounded in local social and health system contexts. The complexity of these factors in Latin America is bound to have an important impact on adequate medical care in RA. Efforts directed to the encouragement of the scientific documentation of these problems in Latin America (LA) are needed.

In order to facilitate the translation and utilization of the existing knowledge about timely and adequate medical treatment of RA into the Latin American context, we need to analyze the literature evidences in the light of their relevance for the local circumstances. The development of a comprehensive conceptual framework aimed to increase understanding of the contextual determinants of the study of the relationship between the different concepts of health care involved can help to map the common or specific characteristics of the research to be undertaken.

## Concepts

### Help-seeking process

The *help-seeking career* has been defined as “a sequence of stages typically passed through by an individual with some real or perceived problem who is on the way to formal treatment, rehabilitation, or perhaps death” [16]. Behavior towards illness and help-seeking are among the most widely used concepts in medical sociology. These concepts range from the initial perception of symptoms, including all relevant experiences and subsequent actions of the patient towards the disease and their coping strategies. Help-seeking includes the need for all kinds of support, medical and nonmedical, to cope with the disease over the so-called illness trajectory [17].

### Illness trajectory

The concept of *illness* goes beyond the biomedical terms of signs, symptoms, and diseases and refers to the cultural dimensions of the disease, particularly to the semiotic (how meaning is created) and phenomenological construction of the symptoms and other forms of expression. Illness is the way that the individuals suffer the alterations of their health in accordance with their biological, psychological, and sociocultural individuality [18].

*The experience of the illness* comprises the perception by the subjects of the progress of their affliction through *time* and actions undertaken to confront it [19]. The lived experience of illness over time makes *illness trajectories*.

Strauss and Glaser [20] coined the term “illness trajectory” to refer “not only to the physiological unfolding of a patient’s disease but to the total organization of work done over that course, plus the impact on those involved with that work and its organization.” This concept revolves around the perception of the patients and considers the impact of the disease on them, together with the responses generated in the course of seeking medical care [20].

Despite the growing body of empirical work devoted to the study of adequate medical care for RA, scientific theory on the integration of social and clinical evidences into a comprehensive model of caring trajectories for the analysis these processes in Latin American context remains underdeveloped [21]. Based on the examination of selected publications on medical care delay, the help-seeking process, and accessibility of RA, this paper is an attempt to begin to address this gap.

### Objectives

1. To identify all qualitative and quantitative research related to help-seeking process and delay in RA medical care in Latin America.
2. To review relevant literature on help-seeking process and delay in RA.
3. To create a comprehensive model integrating the RA medical care processes of help-seeking and delay in a mixed health care system with variable accessibility.

## Material and methods

**Fig. 1:**
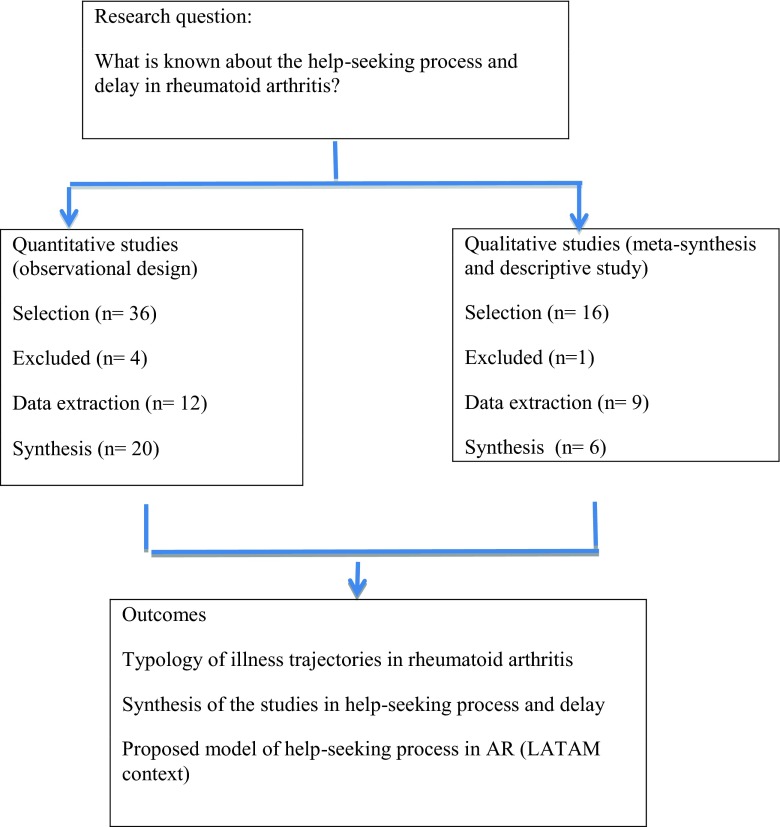
Methods of review. Greenhalgh [22]

Non-systematic literature review of studies with both quantitative and qualitative methodology was conducted. Methodological phases proposed by Greenhalgh [22] were followed:

1. Planning phase: The objective was to assemble a multidisciplinary research team (rheumatologist, medical sociologist, and anthropologist) and to define the research questions for the development of the review.
2. Search phase: The search was conducted in the databases: MEDLINE, EMBASE, LILACS, SOCIAL SCIENCE INDEX, and PSYCOINFO. The key words were help-seeking behavior, rheumatoid arthritis, and delay. We also did hand searches in key Latin American rheumatology journals, books, and theses. The search included qualitative and quantitative studies published in English or Spanish.
3. Mapping phase: In this phase, the key elements of the selected studies (conceptual, theoretical, methodological, and instrumental) were identified and articles meeting the inclusion criteria were reviewed.
4. Synthesis phase: Data relevant to the objectives of this study were synthesized using interpretive synthesis strategy.
5. Recommendation phase: The construction of a conceptual framework of RA patients for LA (Fig. 1).

## Results

Our findings fall into two categories: (1) illness trajectories based on qualitative synthesis, (2) summary and analysis of quantitative studies.

### Illness trajectories based on qualitative synthesis

**Fig. 2:**
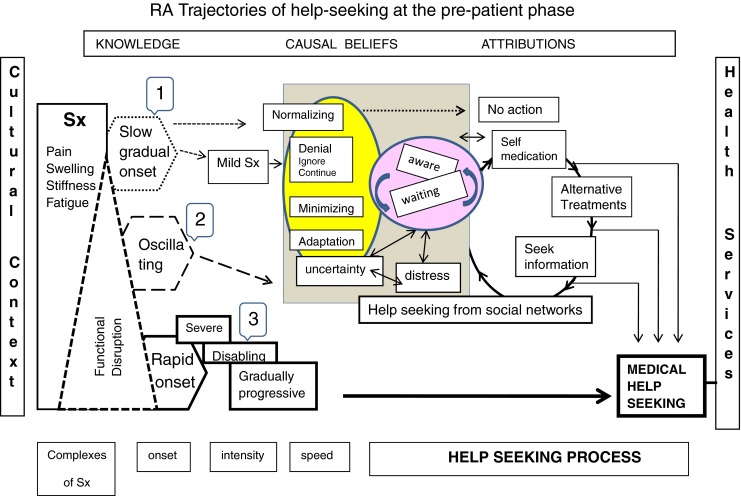
RA trajectories of help-seeking at the pre-patient phase

The empirical findings of the research on help-seeking in RA patients that are documented in the literature confirm the three main types of trajectories for RA proposed by Glasser and Strauss [20]. These trajectories only include the “pre-patient phase” as they begin with the symptom discovery and end when medical care is initiated. Figure 2 depicts a chronological organization (left to right) of the most relevant elements of the three trajectories: Trajectory 1 at top, trajectory 2 at the middle, and trajectory 3 at the bottom (with thick lines). The first element of the trajectories is at the left and includes the symptoms, the type of onset, their intensity, and duration. The way that the illness is experienced within a cultural context (at the left of the figure) as well as the knowledge, beliefs, and attributions (at the top) determine the interplay and pace of following actions graphically summarized in the central and right part of the diagram. The interactions between the experience of illness and the internal reactions of the individual are illustrated in the shadowed box at the middle of the figure (e.g., normalizing, minimizing, adaptation, credibility). Seeking help from the social network takes place all along the trajectory and can delay or facilitate early medical consultation. Lay consultation can begin since the initial perception of the first symptoms in any of the three types of trajectories. Subsequent actions are at the right side of the figure and can range from taking no action to a rapid search for medical consultation. Self-medication, the use of folk or alternative treatments, and seeking information are shown in the figure before seeking medical attention because they have frequently been mentioned as actions that delay the first medical consultation. However, these behaviors can take place at any time: before or after the initiation of formal medical care.

The three trajectories have the following characteristics:

1. *The stable illness trajectories.* The disease is present but is not perceived as such. The symptoms are insidious (gradual), with mild pain due to inflammation. There is no initial impact on daily life activities and there is a minimization of symptoms attributable to quotidian life matters, e.g., old age or type of work. As symptoms are not considered serious, they are normalized [11, 23–25].
2. *The oscillating trajectories*. The disease fluctuates between phases of activity and improvement until the persistence of the symptoms produces disability. The behavior of the individuals is to “wait and see.” Patient minimizes the severity of the disease and experiences uncertainty and distress. This leads to the use of alternative or folk treatments and prompts to seek information and advice from the social network. This trajectory is characterized by having “good days and bad days” [11, 23–25].
3. *The deteriorating trajectories*. Impairment predominates over improvement. Symptoms manifest abruptly, accompanied by increased severity and duration of pain and swelling. The affection of small joints, morning stiffness, and fatigue lead to a significant functional impairment, with an impact on everyday life, emotions, work routine, and family interactions. Even though the patients do not have a favorable response to self-care, they “get used” to pain, but it interferes with their work and daily activities [11, 23–26].

The trajectories 1 and 2 (slow or vague/transitory symptoms) are the ones where patient-related delay has been more commonly found. Symptoms are normalized or not seen as related with RA [27]. In trajectory 3 (rapid onset), the severe pain and the functional impairment accelerate seeking medical attention.

### Summary and analysis of quantitative studies

The literature on medical care in RA places a special emphasis on analyzing the delay in medical help-seeking and the associated variables. However, no quantitative studies have been found in literature measuring the *process* of help-seeking or accessibility to health care services.

The articles distinguish between three types of delay: patient delay, general practitioner (GP) or primary care physicians (PCP) delay, and delay attributable to hospital or specialist care. The latter is further subdivided into delayed diagnosis and delay within the specialist treatment. Further subdivisions are found in the articles but they do not seem to have been found relevant [2, 4, 7, 9, 12, 13, 15].

The quantitative studies reviewed highlighted patient delay and provider or specialist care-related delay. Most studies were conducted in European or North American countries, except for one carried out in Venezuela. No specific studies addressed the systematic study of RA health care accessibility [11, 13, 15, 28].

A decrease in delay time has been documented. Irvine et al. [8] in 1999 noted that the referral periods from a provider to a rheumatologist significantly shortened over a decade. Raza et al. [13] reported in 2011 a variance in delay across several European countries regarding referral to the specialist care, notably if the primary provider had been an orthopedist.

In 2010, van der Linden et al. [2] published a study in which only 31 % of patients sought specialist care before 12 weeks since symptoms onset. According to the article, the main patient-related variables were perception and presentation of symptoms and their impact on everyday life, especially in the workplace. Some studies highlight social barriers to health service utilization such as a low socioeconomic status, living in a rural area, and geographic accessibility [3, 7, 9, 15, 29, 30]. Gender was another factor associated with delay, e.g., there was a larger delay in referral from GPs in female patients [6, 11]. There are also findings that show that the delay was larger when patients were seen by a male physician [6].

In a comparison of service utilization in RA patients from different ethnic groups in a UK city, Kumar et al. [31] also highlights the importance of the cultural background in the help-seeking behavior of a population with the same health service availability. Patient delay was longer in South Asian patients compared with other group. They proposed that these patients’ views of the symptoms “strongly influenced their behavior in response to them.”

Other variables found to be associated to medical care delay were related to primary care and delayed referrals to a specialist. As part of an effort to improve the early diagnosis, the results of an intervention in health services with the implementation of early health care clinics in RA are reported. The result was a decrease in delayed medical care for RA [4].

In most health care systems, PCP acts as the gatekeeper. PCP’s clinical competence for the initial diagnosis is important and is directly related to the delay, continuity, and quality of care. PCP is the one that makes a preliminary diagnosis and refers the patient to the rheumatologist. Relevant studies have revealed differences in diagnostic concordance of RA [32, 33].

When reviewing available data concerning Latin America, we found only one specific study of the delay in a hospital population of Venezuela. Polanco-Rodriguez et al. [34] found the mean between the types of delay to be ranging from 40.5 to 69.7 months (Table 1). The variables associated with the delay were as follows: female, old age, lower education and socioeconomic level, initial examination by an orthopedist, and using public health care services. Over 75 % exhibited erosions in the first 2 years. Another study of poor Hispanic population in Texas reported a delay of 6.9 years from the onset of symptoms to diagnosis [30] (Table 1).

**Table 1:** Summary and analysis of quantitative studies

Author, year (ref)	Objectives	Country	Definition of delay	Results	Variables associated with longer delay
Irvine S et al., 1999 [8]	To study the delay in starting DMARDs in patients with RA, and any changes in medical practice between 1980 and 1997	UK	1. Delay from symptoms onset to referral by a GP and time from referral letter to clinic visit 2. Delays in the use of DMARD treatment	1. Median months: Before 1986, 21 1987–1989, 23 1990–1993, 7 1994–1997, 4 2. Median months: Before 1986, 32 1987–1989, 21 1990–1993, 8 1994–1997, 1	The length of time between symptoms onset and GP referral is dependent on two variables: the delay from symptom onset to consultation with a GP and the time taken for the GP to decide that specialist assessment is required
Hernández-García C et al., 2000 [9]	To study demographic and clinical variables associated with a longer delay in DMARD therapy initiation in a cohort of patients with RA	Spain	1. Time between symptoms onset and first rheumatologist encounter 2. Time between onset of symptoms and first DMARD therapy	Median months (range) 1, 17 (4–74) 2, 19 (7–74)	Delay to DMARD therapy: time between symptoms onset and first rheumatologist visit and years of education. Delay in first visit with rheumatologist: SCJ, age at symptoms onset, home support, labor force status, marital status, and years of education
Palm Ø and Purinszky E, 2005 [11]	Evaluate lag times between disease onset and rheumatological encounter in patients with RA	Norway	1. Time between onset of symptoms and the first encounter with a physician (“patient delay”); 2. Time between the first encounter with a physician and referral to our department (“provider delay”) 3. Time between referral and rheumatological encounter (“hospital delay”)	Median weeks (range) 1, 4 (1–25) 2, 0 (0–34) 3, 4 (0–24)	“Provider delay” was significantly longer in women than in men. The “provider delay” was longer than the “hospital delay” in men and women
Clemente D et al., 2007 [10]	To analyze changes in the lag time to first DMARD prescription since onset of symptoms of RA over the last 2 decades in Spain	Spain	Time between symptoms onset and first DMARD therapy	Median months (range) 14 (6–36)	Delay from symptoms onset to consultation with a GP depending on SJC and accessibility to the health care system Delay of primary care physician for referral to a rheumatologist A significant shortening in the lag time to first DMARD therapy
Kumar K et al., 2007 [12]	To study the delay from the time of symptoms onset to assessment by a rheumatologist in patients with RA and to determine the contributions of patient and physician-related factors to this delay	UK	1. Time taken for patients with symptoms to consult their GP (patient-related factors) 2. Time taken for GPs to refer to a rheumatologist (physician-related factors)	Median weeks (IQR) 1, 12 ( 4–28) 2, 23 ( 12–54)	Patient-related factors leading to a delay in consulting primary care physicians are the principal reasons for the delay in patients with RA being seen by rheumatologists
Feldman DE et al., 2007 [29]	Determine whether patients suspected of having new-onset RA consulted with a rheumatologist, to document any delay in these consultations, and to determine factors associated with prompt consultation	Canada	Time between first diagnostic visit and consultation with the rheumatologist	Median days (IQR) 79 (IQR 28–228)	Older age, lower socioeconomic status, less education, and less access to health services
Kumar K et al., 2010 [31]	Determine the influence of ethnicity on delay	UK	1. Time from the onset of symptoms to a patient’s being assessed in primary care 2. Time from the initial assessment in primary care to a referral to secondary care 3. Time from the referral to secondary care to the patient being seen by a rheumatologist	Median weeks (IQR) 1, 24 (IQR 8–104) 2, 2 (IQR 1–12) 3, 4 (IQR 2–8)	Patient delay was significantly longer in patients of South Asian origin than in other patients Delays at the level of primary and secondary care were not different Four interlinking themes influenced consulting the GP: symptom experience, symptom evaluation, existing ideas, knowledge of RA, and influence of friends and family
van der Linden MP et al., 2010 [2]	Examine the association between delay in assessment by a rheumatologist, rates of joint destruction, and probability of achieving DMARD-free remission in patients with RA	Holland	1. Time from onset of symptoms and a patient’s being seen by a GP (patient delay) 2. Time between the patient’s first assessment by his/her GP and the time when s/he was seen by a rheumatologist (GP delay)	Median weeks (IQR) 1, 2.4 (0.7–7.4) 2, 8.0 (2.7-18.4)	Delay in assessment by a rheumatologist: older age, gradual symptoms onset, involvement of small joints, presence of anti-CCP-2 and IgM-RF, and lower CRP levels Delay in assessment by a rheumatologist leads to a worse disease outcome, as measured by the rate of joint destruction and by achievement of sustained DMARD-free remission
Raza K et al. , 2011 [13]	Quantify delays in assessment of patients with RA across several European countries and to identify whether the principal reasons for delay varied between countries	Germany, UK, Greece, Sweden, Czech Republic, Austria, Poland, and Switzerland	1. Delay from symptoms onset to request to HCP of contact 2. Delay from request to see initial HCP of contact to assessment by that HCP 3. Delay from initial assessment by HCP to referral to a rheumatologist 4. Delay from referral to a rheumatologist to assessment by that rheumatologist	Range (min-max) inter countries weeks 1, 2–22 (Germany-Greece) 2, 1–12 (UK-Greece) 3, 2–12 (UK-Poland) 4, 1–11 (Austria-Germany)	Delay of the initial HCP in referring to a rheumatologist was an important contributor to overall delay. In some centers the initial HCP of contact was frequently an orthopedic surgeon
Jamal S et al. , 2011 [3]	Determine the proportion of patients with RA seen by rheumatologists and treated with DMARD within 3 months of symptoms onset	Canada	1. Time from symptom onset to DMARD initiation 2. Time from RA diagnosis by a rheumatologist to DMARD initiation 3. Time from symptom onset to rheumatologist referral 4. Time from rheumatologist referral to DMARD	Median months (IQR) 1, 6.35 (3.29–12.01) 2, 0 (0.00–0.99) 3, 3.03 (1.02–8.04) 4, 2.01 (1.02–4.01)	Only baseline SJC was found to significantly predict treatment with DMARD within 3 months of symptom onset Younger age and less education influenced the delay in consultation by the patient
Rodriguez-Polanco E et al., 2011 [34]	Estimate the lag time between onset of symptoms, diagnosis, and initiation of DMARD treatment	Venezuela	1. Time between initiation of symptoms and diagnosis of RA 2. Time between onset of symptoms and first consultation with GP 3. Time between first consultation and diagnosis 4. Time between onset of symptoms and initiation of first DMARD 5. Time between diagnosis and initiation of DMARD treatment	Median months (SD) 1, 40.5 (60.3) 2, 16.3 (28.2) 3, 23.9 (48.4) 4, 56.9 (69.7) 5, 13.4 (35.7)	Lower socioeconomic class, lower level of education, first consultation in a public health center, being seen by a GP or an orthopedist as first consultant
van Nies JA et al., 2013 [14]	Assess the motivations and the urgency with which patients with arthralgia seek medical help	Holland	Time between symptoms onset and the first visit to the GP	Median weeks (range) 4.1 (0.9–17.14)	A prolonged delay in seeking help was associated with a gradual onset of symptoms, younger age in women, and the perception that symptoms are not serious
van Nies JA et al., 2013 [4]	Investigated the efficacy of the EARC to decrease the GP delay and consequently reduce the total delay in identifying arthritis	Holland	Time between the first visit to the GP and the first visit to a rheumatologist (delay GP)	Median weeks (range) 8 (2.7–18.4)	Delay was longer before the implementation of EARC, which involved training GPs to recommend an early referral
Villeneuve E et al., 2013 [5]	A systematic literature review to identify effective strategies to reduce delays in the diagnosis and management of IA, in particular RA	UK	1. From to symptoms onset to assessment in primary care 2. From GP to rheumatology referral 3. From rheumatology referral to assessment 4. From rheumatology assessment to commencement of DMARD therapy	Unreported	Lack of patient education on recognizing the symptoms, inadequate GP and health professional training programs and lack of implementation of EARC
Widdifield J et al., 2014 [6]	Estimate the percentage of patients with RA who were seen by a rheumatologist within 3, 6, and 12 months of suspected diagnosis by GP	Canada	Time to be seen by a rheumatologist in patients with RA within 3, 6, and 12 months	3 months, 59 % 6 months, 75 % 12 months, 84 %	The strongest independent with lower frequency of rheumatology visits: patients who lived at remote distances from rheumatologists and male family physicians
De Cock D et al., 2014 [15]	Quantify the different stages of delay before RA treatment in different rheumatology centers and to explore influencing factors	Belgium	1. Patient delay according to the patient^a^ 2. Patient delay according to the GP^b^ 3. GP delay^c^ 4. Rheumatologist delay 1^d^ 5. Rheumatologist delay 2^e^ 6. Total rheumatologist delay^f^ 7. DMARD initiation delay^g^ 8. Total delay^h^	Median weeks (IQR) 1, 10 (4–22) 2, 10 (4–24) 3, 4 (0–13) 4, 6 (3–11) 5, 0 (0–1) 6, 7 (4–12) 7, 1(0–3) 8, 23(14–43)	Total delay: public hospital. Delay were inversely related to disease activity and severity parameters Patient delay was influenced by the intensity and perception of symptoms and contributed most to overall delay
Badley EM et al., 2015 [28]	Examine the per capita rate of visits to rheumatologists as an indicator of access to care for all arthritis and inflammatory arthritis	Canada	1. Rate of visits to GP 2. Rate of visits to rheumatologists	Median (range) rate per 1,000 inhabitants 1, 11.5 (3.3–30.6) 2, 6.9 (1.8–14.8)	Patients living in areas with low access to GP or low SES were less likely to have office visits to rheumatologists
Molina E et al., 2015 [30]	Examine the association of SES and delays in DMARD treatment with clinical measures in RA patients	US	Time between symptoms onset and DMARD initiation	Median years (SD) 6.9 years (SD 9 )	Lower SES, Hispanic origin, public hospitals and distance from the patient’s home to where they received their rheumatologic care
Sørensen J et al., 2015 [7]	Examine the delay in diagnosis of RA, PSA, and AS changed from year 2000 to 2011 (Danish DANBIO registry)	Denmark	Time between onset of symptoms to diagnosis of RA, PSA, and AS	Median months (SD) RA, 23 (41) PSA, 41 (57) AS, 88 (79)	Female patients, older age and lower socioeconomic status Since year 2000, a significant reduction in diagnostic delay was observed in this large cohort

*IQR* interquartile range, *SD* standard deviation, *CRP* C-reactive protein, *DMARD* disease-modifying antirheumatic drugs, *GP* general practitioner, *RA* rheumatoid arthritis, *PSA* psoriatic arthritis, *AS* ankylosing spondylitis, *CCP* anti-cyclic citrullinated peptide, *RF* rheumatoid factor, *HCP* health care professional, *EARC* Early Arthritis Recognition Clinic, *SES* socioeconomic status, *SJC* swollen joint count

^a^Time elapsed between symptoms onset as viewed by the patient and first visit to a GP regarding RA symptoms

^b^Time elapsed between symptoms onset as observed by a GP and first visit to a GP regarding RA symptoms

^c^Time elapsed between first visit to a GP and referral to a rheumatologist

^d^Time elapsed between referral to and first screening by a rheumatologist

^e^Time elapsed between first screening by a rheumatologist and start of treatment

^f^Time elapsed between referral to a rheumatologist and start of treatment

^g^Time elapsed between diagnosis of RA and start of treatment

^h^Time elapsed between symptoms onset and start of treatment

Studies published in Latin America shed some light, albeit indirectly, on the problem of delay. Massardo et al. [35] found a mean delay of 6 years at the moment of the first visit in patients of a private university hospital in Chile. Public hospital patients had a shorter time lag between the onset of symptoms to the date of the first consultation (3 years) and the patients also had fewer years of formal education. The authors suggest that the difference in time lag from disease onset to the first consultation could have been due to the local patterns of referral. In 2012, Acevedo Vásquez [36] reported in Peru a delay in the diagnosis of RA of 2.29 ± 5.24 years in patients seen within the social security system.

In 2001, Kaliski et al. [37] published a study on RA in Mapuche aborigines in Chile. One hundred and six patients from a public specialist hospital were included, most of them (87.7 %) from rural areas. The mean delay in diagnosis was 4.4 years, 46.9 % had functional class III/IV at the beginning.

A study looked at social and demographic characteristics of RA patients in the province of Córdoba, Argentina, by administering a questionnaire to patients who accessed to the health care services, both public and private. They found that the diagnosis was made later in men; 39.1 % had fewer than 5 years of evolution of the disease, 37.5 % were attending to a public hospital; 80 % were from urban areas. Only 29.7 % had paid work and 69 % were economically dependent on another person [38]. Another study of hospital population in Argentina reported a delay of 12 months from first symptoms to a consultation with a rheumatologist [39].

With regard to the public awareness concerning RA, in Argentina, a representative study of the general population from different areas of the country **(***n* = 24,324**)** revealed that 29 % did not know that rheumatic diseases could affect children and young people, and 19 % did not know that these diseases could cause deformities and disabilities [40].

## Discussion

The help-seeking process of RA poses important challenges: illness is more than the clinical manifestations of the disease and the response to illness is a socially constructed process coherent with the meanings that the patient and his context give to the symptoms experience. The knowledge about the symptoms as perceived by the patients is still insufficient. The illness has also been reduced to the symptoms alone and not to the whole illness experience within their sociocultural contexts [41].

The trajectories and the health service utilization pathways are linked to the specific characteristics of the health and social systems that go from the micro to the macro level determinants. Hence, the theoretical framework that we propose here has to be understood in the light of each context and must also take into account the individual characteristics of each patient and his/her disease onset and evolution, i.e., the illness trajectory. One of the more relevant limitations that the illness trajectory concept has had on medical care utilization research on RA is that “the illness trajectory concept has the value of analytically ordering the events that occur over a sickness episode…but it cannot explain the link between such events” [21]. It is not only that social interaction has a descriptive important influence on the help-seeking process that will eventually lead to a diagnosis but they can also explain why this happens.

It has been reported that individuals with RA have little knowledge of the disease and resort to their immediate social network to get information and help [24]. This is in line with the results of the Argentinean survey and the English study, which describe a limited knowledge of the rheumatic diseases [27, 40]. The social network members do not always give support or speed up medical care but extend the time to seek medical help. On one hand, in RA, rapid onset episodes or the presence of severe or disabling symptoms activate prompt help-seeking both from the network and from the doctor [24]. Similar to many other studies, the role of social factors as mediators of medical help-seeking decreases with extreme situations: symptom severity and disabling progression [42]. On the other hand, Kumar et al. found that compared with British patients, the South Asian social networks delayed RA patients’ medical attention because the latter extensively discussed their problem with their family and friends from both, the UK and from their native countries. This patients and social network behavior is not surprising as they belong to a collectivist culture whereas England is an individualistic society [31].

### Model of help-seeking process in RA in LA

The results of our review show that in RA medical care utilization research, the theories and measurements of the constructs of illness trajectories, help-seeking, and accessibility are not integrated. Clinical and biomedical research has produced an important body of knowledge about the ideal medical treatment of RA, especially when the disease is identified early. However, medical treatment of RA involves the contact between the individuals and the health services and thus their study requires theories and frameworks that allow for the understanding of these relationships as complex social phenomena. Many of the social and contextual aspects have been studied with isolated traditional social variables that are only the tip of the iceberg of complex social realities that need to be considered and connected. There is a need of a multilevel perspective (e.g., the individuals, the social environment, the health system), together with the examination of the links between the different levels and dimensions of the problem. Many aspects of the illness experience, of the help-seeking process, and of access and delay of RA medical care have been studied, but it is not easy to locate or to explain the research problems and the different results obtained within a broad theoretical frame of reference. Furthermore, the concept of RA help-seeking is frequently used as a synonym of medical help-seeking. This is an example of the conceptual confusions due to the lack of clarity of the theories underlying health services utilization and medical care research.

The research in RA medical care utilization in different contexts other than very few European countries and Canada is almost nonexistent. Most of the literature covers the study of the medical care service utilization of RA patients in countries with universal public health care systems that covers all population. Problems such as health care inequity and cultural competence, availability, affordability, and accessibility of primary care physicians and specialists have not been specifically studied within a comprehensive health service framework. They do not seem to be relevant problems in the research agenda. However, for most of developing countries, this needs to be a priority. They have a completely different social context and most of the health services are insufficient, inaccessible, fragmented, limited, and inequitable in both quantity and quality. If research on RA service utilization is to be undertaken in these countries, there is a need for a comprehensive conceptual framework than can enable researchers to integrate the study of the problems within broad theoretical and methodological perspectives.

**Fig. 3:**
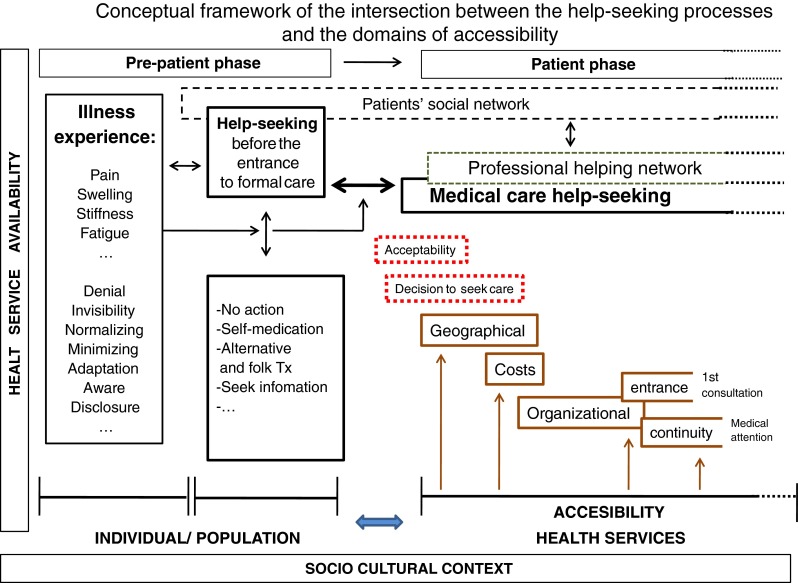
Conceptual framework of the intersection between the help-seeking processes and the domains of accessibility

In order to have a more clear perspective of the problems that fall under the broad concept of health service utilization of RA patients, we propose a conceptual framework that graphically synthesizes the intersection between the *help-seeking processes* and the domains of the concept of *accessibility* (Fig. 3). There is much confusion between these two concepts mostly because they partly overlap in a time sequence and because they are commonly used in two fields with their own definitions: the social sciences and health service research.

On the one hand, the concept of *accessibility* (or access) is restricted to formal health services and pertains to the field of health services utilization. Under a broad perspective, Donabedian [43] includes service accessibility as part of quality of health care. On the other hand, the notion of *help-seeking* belongs to the social sciences and covers all stages and process of seeking any kind of help useful to meet the health needs [43]. However, this process has a sequence in time and is dynamic in nature as many elements interact simultaneously (e.g., symptom perception, individual behavior, and family advice).

The two main phases of the *help-seeking process* are depicted at the top of the diagram: the *pre-patient* and the *patient phase*. The characteristics of the population are at the left side and those of the health services are at the right. The domains of health service *accessibility* are specified at the right side and comprises the three (not strictly) sequential aspects of accessibility: *geographical*, *economic* (costs), and *organizational* accessibility. The latter is subdivided into two: the entry process and the continuity of care. As the patient phase includes what happens from the initial contact with the health services, the accessibility problems coincide with the second part of the help-seeking, namely *medical care help-seeking*.

Following McKinlay [42], the pre-patient phase comprises the range of behavior and contacts that occur prior to encountering professional, formal health care. Hence, the notion of help-seeking does not stop or start at the entrance to professional health services and it is not restricted neither to formal nor medical health services, as it is commonly assumed. In fact, as shown in Fig. 2, the transition from the pre-patient phase to the patient phase is an important step in help-seeking because it is the point at which the two different social networks get together. Then the *professional helping network* begins to exert greater social control than the social network. The notion of “social network” refers to “that set of contacts with relatives, friends, neighbors, etc., through which individuals maintain a social identity and receive emotional support, material aid, services and information and develop social contacts.” The functions of the “social network” activated by the illness experience do not stop after medical care starts, as it is part of the broader permanent web of interpersonal relations and social capital of the individual.

Access is not synonym of availability or use of services. The latter is the evidence of access, and *availability* is the mere existence of the service. Health service availability placed at the left side is the first element of medical help-seeking and of accessibility to formal health services. *Accessibility* is placed at the second half of the figure, as it is the result of the degree of fit between the characteristics of the individual or population and those of the health services [43].

After the individual decides to ask for available medical care, he faces *geographical* accessibility problems because s/he has to be able to travel and has to have the means to do it. The next type of accessibility to be faced are all types of *costs* (monetary and opportunity cost): problems such as transportation, medical fees, waiting times, service interruptions, medicines, lab tests, time out of work (e.g., younger population faces longer delays to specialized RA care because they might have less time than the retired or the elderly to seek medical care) [43]. The components of the *organizational* accessibility are the *entry* to the system (e.g., if they struggle to get an appointment or they have to wait long between they are given an appointment and the appointment itself) and the *continuity* of care, i.e. subsequent service utilization.

We propose this conceptual integrated framework for the study of RA in order to join the elements of the pre-patient help-seeking to the medical care help-seeking (or patient phase) together with the specification of the different types of accessibility problems of the social and health services contexts. However, this framework can also be used for any other type of rheumatologic diseases or can be linked to other diseases such as cancer [44, 45]. The experience of chronicity in rheumatologic diseases also needs to be considered. Chronicity can be seen as an irreversible event in the experience of a disease. A relationship between time and discomfort, without the chance of going back to the previous stages of life, foments irreversibility, which is experienced with anguish and sadness. Suffering becomes part of the patient.

The cultural context of this model should also be considered before implementation. This requires incorporating elements such as medical pluralism, which refers to the mix of health care systems with the processes that the individuals undergo to attend their discomfort or suffering. The combination or use of different care systems allows individuals to cover the diverse expressions of their illness, which vary according to many factors such as severity or chronicity.

The study about the problems for the ideal medical care utilization for RA is also a challenge because it is located at the intersection of clinical, sociomedical, epidemiological, and health care research. Research efforts are needed to analyze and integrate the accumulated knowledge on this subject, to facilitate the understanding of a comprehensive perspective of this phenomenon, and for the identification of the research areas that are needed in order to improve the response of health care services to RA patients needs.
